# The Influence of Green Surface Modification of Oil Palm Mesocarp Fiber by Superheated Steam on the Mechanical Properties and Dimensional Stability of Oil Palm Mesocarp Fiber/Poly(butylene succinate) Biocomposite

**DOI:** 10.3390/ijms150915344

**Published:** 2014-08-29

**Authors:** Yoon Yee Then, Nor Azowa Ibrahim, Norhazlin Zainuddin, Hidayah Ariffin, Wan Md Zin Wan Yunus, Buong Woei Chieng

**Affiliations:** 1 Department of Chemistry, Faculty of Science, Universiti Putra Malaysia, 43400 UPM Serdang, Selangor, Malaysia; E-Mails: norhazlin@upm.edu.my (N.Z.); chieng891@gmail.com (B.W.C.); 2 Department of Bioprocess Technology, Faculty of Biotechnology and Biomolecular Sciences, Universiti Putra Malaysia, 43400 UPM Serdang, Selangor, Malaysia; E-Mail: hidayah@upm.edu.my; 3 Department of Chemistry, Centre For Defence Foundation Studies, National Defence University of Malaysia, Sungai Besi Camp, 57000 Kuala Lumpur, Federal Territory of Kuala Lumpur, Malaysia; E-Mail: wanmdzin@upnm.edu.my

**Keywords:** biocomposite, dimensional stability, mechanical properties, oil palm mesocarp fiber, poly(butylene succinate), superheated steam

## Abstract

In this paper, superheated steam (SHS) was used as cost effective and green processing technique to modify oil palm mesocarp fiber (OPMF) for biocomposite applications. The purpose of this modification was to promote the adhesion between fiber and thermoplastic. The modification was carried out in a SHS oven at various temperature (200–230 °C) and time (30–120 min) under normal atmospheric pressure. The biocomposites from SHS-treated OPMFs and poly(butylene succinate) (PBS) at a weight ratio of 70:30 were prepared by melt blending technique. The mechanical properties and dimensional stability of the biocomposites were evaluated. This study showed that the SHS treatment increased the roughness of the fiber surface due to the removal of surface impurities and hemicellulose. The tensile, flexural and impact properties, as well as dimensional stability of the biocomposites were markedly enhanced by the presence of SHS-treated OPMF. Scanning electron microscopy analysis showed improvement of interfacial adhesion between PBS and SHS-treated OPMF. This work demonstrated that SHS could be used as an eco-friendly and sustainable processing method for modification of OPMF in biocomposite fabrication.

## 1. Introduction

Natural fibers as filler in biocomposites have received substantial interest from researchers because they have many significant advantages over synthetic fibers. They are abundantly available at low cost, environmentally friendly, renewable and biodegradable, as well as have low density [1]. For example, fibers of althaea [2], artichoke [3], arundo [1,4], bamboo [5], borassus fruit [6], coir [7,8,9], curaua [10], ferula [11], jute [12,13], kenaf [14,15,16], oil palm [17,18,19,20,21,22,23,24] and sansevieria [25] have been utilized for biocomposite applications.

Malaysia is one of the major oil palm cultivation countries in the world. Consequently, large amounts of oil palm biomass are left behind in the palm oil mill [17]. This includes oil palm mesocarp fiber (OPMF), a lignocellulose fiber obtained from the oil palm fruit after the oil extraction process [18]. It is a renewable material and can be obtained at a very low cost directly from the oil palm mill throughout the year as by-product of palm oil production. It is normally burnt as a boiler fuel to generate steam and electricity for the palm oil mill [19]. Recently, our research group has successfully compounded it with thermoplastics of poly(lactic acid) (PLA), poly(butylene succinate) (PBS) or PLA/poly(caprolactone) blend to produce biodegradable biocomposites [20,21,22]. This type of biocomposite shows numerous advantages such as light weight, low cost, biodegradable, and exhibits reasonable strength and stiffness [5].

PBS is a biodegradable thermoplastic synthesized by the polycondensation of 1,4-butanediol and succinic acid [26]. It is a bio-based thermoplastic as 1,4-butanediol and succinic acid can be produced by fermentation [27]. It can be degraded by microorganisms in compost, soil, and seawater [14]. The mechanical properties of PBS are reported similar to those of the conventional thermoplastics such as polyethylene and polypropylene which are non-biodegradable [12]. Additionally, the price of PBS is lower than those of other commercially available biodegradable thermoplastics such as PLA and poly(hydroxylbutyrate) (PHB) [7]. These advantages make it an attractive alternative compared to those of other biodegradable and non-biodegradable thermoplastics.

In previous work, biocomposites consisting of PBS and various content of OPMF were successfully fabricated by melt blending technique [21]. However, these biocomposites exhibited low tensile, flexural and impact properties resulting from the poor interfacial adhesion between hydrophilic OPMF and hydrophobic PBS. This problem can be overcome via several approaches such as introducing a compatibilizer or coupling agent into the biocomposite [5,8,10,14,28,29], and modifying the fiber surface properties via NaOH treatment [6,7,8,12,15,18], chemical grafting [20] or bleaching [16]. These modifiers or modifications improved the biocomposite properties but most of them are not eco-friendly, toxic or/and expensive. Therefore, finding a cheap yet environmentally friendly modification method is highly recommended.

Recently, superheated steam (SHS) has been utilized to modify lignocellulosic materials for various applications. SHS is dry steam produced by boiling wet steam with additional heat at a given pressure [30]. It is a simple, cheap and eco-friendly technique for surface modification of fiber as water is the only reactant used in the process. Therefore, it will be cost effective for large scale purposes [17]. Currently, SHS has been used to treat fiber for bio-fuel and bio-sugar productions [17,23,31]. Those studies revealed that at high steam temperature, hemicellulose would be first degraded as it is less thermally stable in comparison to those of cellulose and lignin. The removal of hemicellulose subsequently increases the accessibility of cellulose to enzymatic hydrolysis and resulted in a higher sugar yields. On the other hand, it is reported that there is an increase in fiber hydrophobicity and thermal stability after removal of hemicellulose [28]. Those properties of fiber are beneficial in biocomposite production. Therefore, there is no doubt that SHS can be used as an alternative treatment method for modification of fiber in biocomposite production. To date, there is no study on the utilization of SHS-treated OPMF in biocomposite fabrication has been reported.

In the present work, OPMF was treated with SHS aiming at modification of fiber’s surface. The treated fiber was characterized by Fourier transform infrared (FTIR) spectroscopy and scanning electron microscopy (SEM). The effect of SHS-treated OPMFs on the mechanical properties and dimensional stability of OPMF/PBS biocomposite was reported.

## 2. Results and Discussion

### 2.1. Characterizations of Untreated OPMF and SHSOPMF

#### 2.1.1. FTIR Spectroscopy

**Figure 1:**
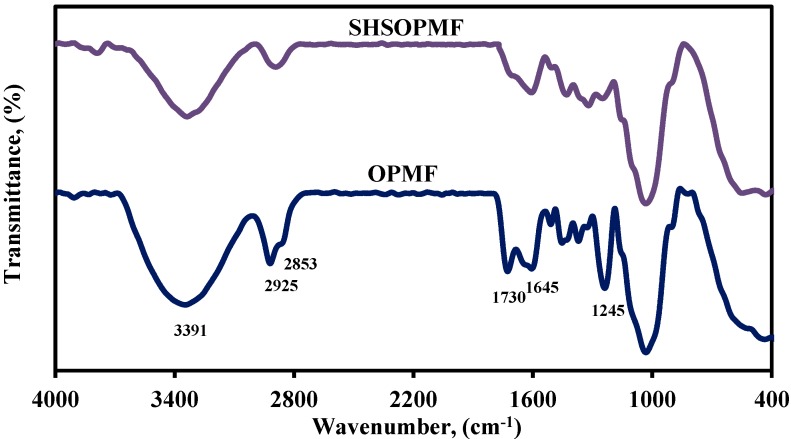
FTIR spectra of OPMF and SHSOPMF.

FTIR spectroscopy was employed to identify the functional groups and chemical components of fiber. The OPMF treated at temperature of 220 °C for 60 min (hereafter referred as SHSOPMF) was used as a representative for SHS-treated OPMFs because OPMF treated at these conditions yielded biocomposites with optimum tensile properties (discussed in Section 2.2.1). The FTIR spectra of OPMF and SHSOPMF are illustrated in Figure 1. The significant differences between these two spectra are clearly seen at peaks of 1730, and 1245 cm^−1^, assigned to C=O stretching of ester and carboxyl groups in hemicellulose, and C–O stretching of acetyl group in hemicellulose and lignin, respectively [6]. The absorbance of these peaks on the SHSOPMF spectrum was drastically reduced as compared to that of the OPMF spectrum. This indicates that SHS treatment removed hemicellulose and lignin partially. A similar result was also reported by other researchers [15,17,24]. In addition, the absorbance of peaks at 2925 and 2853 cm^−1^, corresponding to C–H stretching of cellulose and hemicellulose in the SHSOPMF spectrum is also reduced due to the removal of hemicellulose [32]. It is also seen that the hydrophilicity of SHSOPMF was reduced in comparison to that of OPMF as verified by a decrease in the absorbance of peaks at 3391 and 1645 cm^−1^, corresponding to OH stretching and OH bending of absorbed water, respectively [32].

#### 2.1.2. Surface Morphology

**Figure 2:**
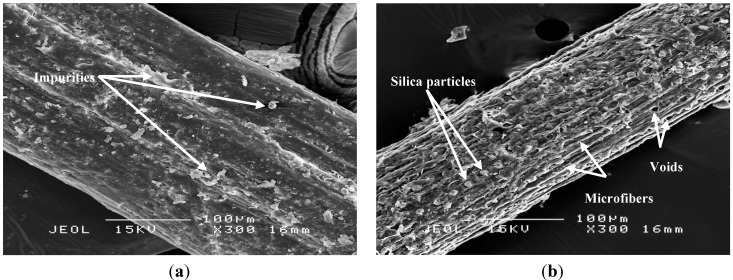
SEM micrographs of (**a**) OPMF and (**b**) SHSOPMF.

The surface morphologies of OPMF and SHSOPMF are determined by SEM analysis as shown in Figure 2. The untreated OPMF (Figure 2a) exhibits a smooth surface with some impurities such as waxy and oily substances covering its surface. However, after SHS treatment, those impurities are removed and the surface of the fiber becomes rougher and textured (Figure 2b). This observation is similar to those described by other researchers [15,17,24]. Moreover, the silica particles and microfibers can be clearly seen on the fiber surface. The micrograph of SHSOPMF also shows the presence of voids which are due to the removal of silica particles by SHS treatment. These voids are beneficial in biocomposite production as it may facilitate the penetration of thermoplastic into the fiber and therefore create a good mechanical interlocking between the thermoplastic and fiber [16]. Furthermore, the presence of microfibers may also increase its surface area of contact with the thermoplastic, leading to stronger interfacial adhesion and producing biocomposites with enhanced mechanical properties [13].

The FTIR and SEM analyses have shown the ability of SHS treatment in removing the impurities and hemicellulose from the OPMF by producing fiber with rough surface morphology. Similar observation is also reported by Ibrahim *et al.* [15] in NaOH-treated kenaf fiber. However, SHS treatment is more environmentally benign relative to NaOH treatment as no toxic solvent or chemical is used during the modification process. Furthermore, the dry modification process of SHS is also an advantage, as no drying step is needed after treatment. The treated fiber can be directly utilized without further drying in biocomposite fabrication. Consequently, SHS can be used as an alternative method to NaOH for modification of fiber.

### 2.2. Characterizations of Untreated OPMF/PBS and SHS Treated OPMF/PBS Biocomposites

The main factors influencing the degree of modification of fiber during SHS treatment are temperature and time of treatment. Therefore, the effect of these two parameters is directly accessed by tensile properties measurement of its corresponding biocomposites.

#### 2.2.1. Tensile Properties

The effectiveness of SHS-treated OPMF as reinforcement in biocomposites are assessed by comparing the tensile results of SHS-treated OPMF/PBS biocomposites and untreated OPMF/PBS biocomposites. Table 1 presents the tensile strength (TS), tensile modulus (TM), and elongation at break (EB) of untreated OPMF/PBS and SHS treated OPMF/PBS biocomposites. The TS, TM and EB of neat PBS are reported to be 37.31 and 248.90 MPa, and 470%, respectively [21]. The addition of 70 wt % of OPMF to PBS has reduced its TS, TM and EB.

**Table 1:** Tensile properties of untreated OPMF/PBS and SHS-treated OPMF/PBS biocomposites.

Biocomposite	Temperature (°C)	Time (min)	Tensile Strength (MPa)	Tensile Modulus (MPa)	Elongation at Break (%)
OPMF/PBS	-	-	13.86 ± * 0.73	94.80 ± 7.60	2.50 ± 0.29
SHS treated OPMF/PBS	200	60	16.06 ± 0.58	111.82 ± 8.51	2.28 ± 0.29
	210	60	17.72 ± 0.60	122.30 ± 3.21	2.65 ± 0.31
	220	30	16.26 ±1.00	128.00 ± 15.85	2.68 ± 0.25
		60	19.42 ± 0.45	552.50 ± 42.27	3.15 ± 0.35
		90	19.64 ± 1.27	261.60 ± 41.30	2.87 ± 0.22
		120	20.57 ± 1.26	122.67 ± 16.17	3.06 ± 0.28
	230	60	17.46 ± 0.45	571.43 ± 19.27	2.90 ± 0.42

* ± = standard deviation.

As shown in Table 1, at 60 min treatment time, the TS and EB of the SHS-treated OPMF/PBS biocomposites increase with increasing treatment temperature from 200 to 220 °C and then decrease at 230 °C. At 220 °C, it is found that improvement of 40% and 26% are recorded for TS and EB, respectively as compared to that of untreated OPMF. An increasing trend is also observed for TM. It increases with increasing treatment temperature of up to 220 °C and levels off at 230 °C. At 220 °C, the TM is increased by 483% in comparison to that of untreated OPMF. It is obvious that at 60 min treatment time, the OPMF treated at temperature of 220 °C yielded biocomposites with optimum TS, TM and EB as compared to those of other treatment temperature and untreated OPMF. Therefore, this temperature was selected to conduct further experiments in order to determine the optimum time for this treatment.

The TS of SHS-treated OPMF/PBS biocomposites increases with increasing treatment time of up to 60 min and remains nearly constant until 120 min. However, the highest values of TM and EB are observed at 60 min treatment time and then decrease at higher treatment time. From the tensile test result, it can be concluded that OPMF treated at 220 °C and 60 min produced biocomposites with optimum TS, TM and EB in comparison to those of other treatments temperature and time, as well as untreated OPMF.

The increment in tensile properties of SHS-treated OPMF/PBS biocomposite may be attributed to the fact that SHS treatment improves the adhesive characteristic of the OPMF surface by removing impurities covering the surface of fiber [13]. This normally gives better bonding to thermoplastic via mechanical interlocking mechanisms. Besides, the increase in surface hydrophobicity of SHS-treated OPMF also leads to better compatibility toward hydrophobic PBS and improves the biocomposite tensile properties [22]. The percentage of increment in tensile properties (TS and TM) is relatively small (<28%) at low temperature (200–210 °C) and short treatment time (30 min). At these conditions, limited amount of impurities and hemicellulose have been removed as hemicellulose only starts to degrade at temperatures around 220 °C [33]. The adhesion between fiber and PBS might be improved but still inadequate to transfer load effectively from thermoplastic to fiber. Hence little improvement is observed in the tensile properties of the SHS treated OPMF/PBS biocomposites. Meanwhile at high temperature (230 °C) and long treatment time (90–120 min), degradation of hemicellulose may occur rapidly by liberation of large amount of acetic acid. This acid may accumulate on the fiber surface and accelerate the degradation process of cellulose. It is reported previously that cellulose in fiber contributes considerably to its mechanical strength. As a result, degradation of cellulose leads to the loss of mechanical strength in fiber and subsequently reduces the biocomposite tensile properties [24].

#### 2.2.2. Surface Morphology

**Figure 3:**
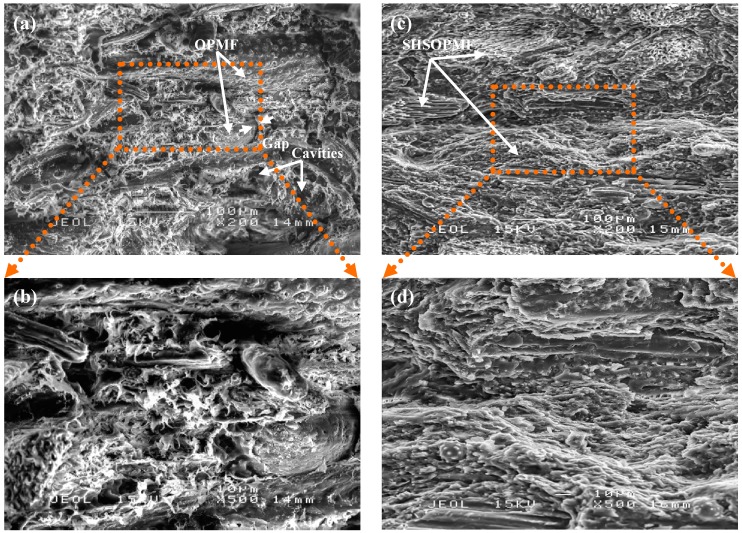
SEM micrographs of untreated OPMF/PBS (**a**),(**b**) and SHSOPMF/PBS (**c**),(**d**) biocomposites.

The tensile fracture surface of SHSOPMF/PBS biocomposite is studied under scanning electron microscope in order to study the adhesion between SHSOPMF and PBS. Figure 3 shows the scanning electron micrographs of tensile fracture surfaces of untreated OPMF/PBS and SHSOPMF/PBS biocomposites. As shown in Figure 3a,b, the untreated OPMF and PBS remained as two distinguishable phases due to incompatibility between hydrophilic fiber and hydrophobic thermoplastic [21]. The fiber is packed loosely with PBS with a visible gap at their interface region. This indicates that the adhesion between these two phases is poor. Moreover, cavities can also be seen due to the fiber pulled out from the matrix.

However, after SHS treatment, there is a notable change in tensile fracture surface morphology of SHSOPMF/PBS biocomposite as shown in Figure 3c,d. The loosely packed structure of untreated OPMF/PBS biocomposite has transformed into a compact solid-like structure in SHSOPMF/PBS biocomposite. Furthermore, no visible gap can be seen at the fiber/thermoplastic interface region, indicating the interfacial adhesion between SHSOPMF and PBS is considerably improved. It has been reported that lignin is softening at high steam temperature (160–190 °C), caused it to flow to the fiber surface and then re-solidify upon cooling [34]. The presence of lignin on the fiber surface (lignin-rich surface) can induce fiber hydrophobicity as lignin is relatively more hydrophobic than cellulose and hemicellulose. This in turn increased the compatibility of SHSOPMF and hydrophobic PBS. In addition, the degraded products of hemicellulose such as monosaccharide may also be present on the fiber surface after treatment. This degraded product can act as an adhesive to bind the fiber and PBS together during compounding. This is clearly manifested by the formation of closely packed structures in the tensile fracture surface of SHSOPMF/PBS biocomposite. This also explains the improvement in tensile properties of the SHSOPMF/PBS biocomposite.

#### 2.2.3. Flexural and Impact Properties

The results of flexural and impact tests of untreated OPMF/PBS and SHSOPMF/PBS biocomposites are shown in Table 2. It is apparent that the flexural strength and modulus, as well as impact strength of SHSOPMF/PBS biocomposite are higher than that of untreated OPMF/PBS biocomposite. The flexural strength, flexural modulus and impact strength of OPMF/PBS biocomposite are 27.26, 2191.00 MPa and 65.75 J/m respectively. These values increased to 32.56, 3180.00 MPa and 74.70 J/m for SHSOPMF/PBS biocomposite, showing the increment of 19%, 45% and 14%, respectively. The increment in flexural and impact properties of SHSOPMF/PBS biocomposite results from the improved SHSOPMF/PBS interfacial adhesion, leading to biocomposites with better bending and crack propagation resistance.

**Table 2:** Flexural strength, flexural modulus and impact strength of untreated OPMF/PBS and SHSOPMF/PBS biocomposites.

Biocomposite	Flexural Strength, (MPa)	Flexural Modulus, (MPa)	Impact Strength, (J/m)
OPMF/PBS	27.26 ± * 0.99	2191.00 ± 96.00	65.75 ± 3.08
SHSOPMF/PBS	32.56 ± 0.48	3180.00 ± 50.00	74.70 ± 0.54

* ± = standard deviation.

#### 2.2.4. Dimensional Stability

**Figure 4:**
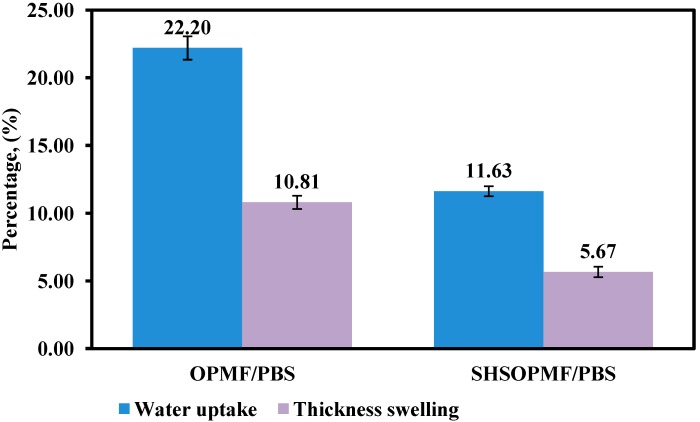
The water uptake and thickness swelling of untreated OPMF/PBS and SHSOPMF/PBS biocomposites.

Water absorption is one of the major concerns in fiber/thermoplastic biocomposites for industrial application, as natural fiber tends to absorb moisture and swell when exposed to water or humid environments [9]. For this reason, the water uptake and thickness swelling tests are conducted on the prepared biocomposites. The water uptake and thickness swelling of untreated OPMF/PBS and SHSOPMF/PBS biocomposites after 24 h of immersion in water are shown in Figure 4. Both biocomposites show increase in water uptake and thickness swelling after 24 h of immersion in water, attributed to the presence of hydroxyl groups in fiber which induced moisture absorption [9]. However, SHSOPMF/PBS biocomposite shows lower percentages of water uptake and thickness swelling than that of untreated OPMF/PBS biocomposite.

As illustrated in Figure 4, the percentage of water uptake of SHSOPMF/PBS biocomposite (11.63%) is reduced by 48% in comparison to that of untreated OPMF/PBS biocomposite (22.20%). This results from the decrease in the amount of hydroxyl groups accessible to water absorption in SHSOPMF as hemicellulose is removed partly by SHS treatment. In addition, the migration of lignin to the fiber surface might provide a protective layer that limits the diffusion of water into the fiber, hence reducing water uptake. This finding is in good agreement with a previous report by Nam *et al.* [27] who studied the water absorption of surface-treated jute fiber/PBS composites. The percentage of thickness swelling of untreated OPMF/PBS and SHSOPMF/PBS biocomposites are 10.81% and 5.67%, respectively. This shows a reduction of 48% in thickness swelling for the SHSOPMF/PBS biocomposite. This is attributed to low water uptake of SHSOPMF/PBS biocomposite as thickness swelling is directly proportional to the amount of water absorbed by the biocomposite.

## 3. Experimental Section

### 3.1. Materials

OPMF was collected from FELDA Serting Hilir Oil Palm Mill, Jempol, Malaysia. The OPMF was washed by soaking in distilled water for 24 h then rinsed with hot water (60 °C) and acetone prior to drying at 60 °C in an oven. This process was carried out to remove dirt adhered to the fiber surface. The dried fiber was then ground, sieved into size of 150–300 μm and stored in a sealed polyethylene bag. PBS, under the trade name of BIONOLLE 1903MD was purchased from Showa Denko, Tokyo, Japan. It has a density of 1.26 g/cm^3^ and melting point of ~115 °C.

### 3.2. Modification of OPMF by SHS Treatment

Modification of OPMF was carried out in a superheated steam oven (Model QF-5200C, Naomoto Corporation, Osaka, Japan) under normal atmospheric pressure following the method described by Ahamad Nordin *et al.* [23]. Regular tap water was used to produce the SHS. Prior to the treatment, OPMF was dried in an oven at 60 °C. The dried OPMF was then treated in a SHS oven at temperature of 200, 210, 220 and 230 °C for 60 min. The experimental procedures were as follows: First, the SHS oven was turned on. The temperature and steam flow rate were set and allowed to reach a steady state. Next, about 20 g of OPMF was uniformly poured on the aluminium foil tray (10 × 12 × 1 cm^3^). It was then put into the heating chamber of the SHS oven under the designated conditions. Once treatment was completed, fiber was removed immediately from the heating chamber, cooled in a desiccator and finally stored in the sealed polyethylene bag. The OPMF-treated at 220 °C gave optimum tensile properties of the biocomposite in comparison to those of other treatment temperatures and untreated OPMF. Therefore this temperature was selected to conduct further experiments with the same procedures at different treatment times (30, 90 or 120 min) in order to determine the best treatment time.

### 3.3. Fabrication of Biocomposites

PBS, OPMF and SHS-treated OPMFs were oven-dried at 60 °C prior to processing. Biocomposites were fabricated by melt blending of PBS and OPMF or SHS-treated OPMFs in a Brabender internal mixer at 120 °C with a rotor speed of 50 rpm. The ratio of OPMF/PBS was fixed at 70/30 as this is the maximum loading of fiber that can be loaded into this system and it showed the highest value of flexural modulus [5]. In brief, PBS pellets were first melted in the Brabender mixing chamber for 2 min. Next, fiber was added slowly into the mixing chamber and continued mixing for another 13 min. The compounded material was compressed into sheets with thickness of 1 and 3 mm by hydraulic hot-press at temperature of 120 °C under pressure of 150 kg/cm^2^ for 5 min, followed by cold pressing at 30 °C for 5 min.

### 3.4. FTIR Spectroscopy

The functional groups and chemical components of OPMF and SHSOPMF were identified by a Perkin Elmer (Waltham, MA, USA) Spectrum 100 series Spectrophotometer equipped with attenuated total reflectance (ATR). The FTIR spectra of the samples were recorded in the range of wavenumber of 400–4000 cm^−1^.

### 3.5. Mechanical Properties Measurement

Tensile test was carried out on the biocomposites by using a Universal Testing Machine, Instron (Buckinghamshire, UK) model 4302 equipped with a 1 kN load cell. The test specimens were cut from 1 mm sheet according to ASTM D638-5. The test was conducted at 25 °C with a crosshead speed of 5 mm/min. The results were expressed in term of tensile strength, tensile modulus and elongation at break.

A three points bending test was conducted on the biocomposites by using a Universal Testing Machine, Instron (Buckinghamshire, UK) model 4302 equipped with a 1 kN load cell, as described in ASTM D790. The test specimens were cut into the dimension size of 127.0 × 12.7 × 3.0 mm^3^. The test was conducted at 25 °C with a crosshead speed of 1.3 mm/min and span length of 48 mm. The results were expressed in term of flexural strength and flexural modulus.

Un-notched Izod impact test was carried out on the biocomposites by using an IZOD (Mumbai, India) Impact Tester equipped with a 7.5 J pendulum at 25 °C, based on ASTM D256. The test specimens were cut into dimension size of 64.0 × 12.7 × 3.0 mm^3^. The impact strength (J/m) was calculated by dividing the energy (J) obtained with the thickness (m) of specimen.

All the above tests were done on five specimens for each formulation. The average values and standard deviations were reported.

### 3.6. Scanning Electron Microscopy

The scanning electron micrographs of OPMF, SHSOPMF and tensile fracture surfaces of biocomposites were recorded by using a JEOL (Tokyo, Japan) JSM-6400 scanning electron microscope operated at 15 kV accelerating voltage. The oven-dried samples were placed on the metal holder and coated with gold by a Bio-rad (Hercules, CA, USA) coating system for 3 min to ensure good conductivity prior to analysis.

### 3.7. Dimensional Stability Measurement

Water uptake and thickness swelling tests were conducted according to ASTM D570 and European Standard EN 317 (1993), respectively. Samples with dimension of 10.0 × 10.0 × 1.0 mm^3^ were cut from the sample sheets and used for testing. Prior to testing, the samples were oven-dried at 60 °C until a constant weight was obtained. The initial weight (*W*_0_) and thickness (*T*_0_) of the dried samples were measured using a microbalance and caliper, respectively. The samples were then immersed into the distilled water for 24 h at 25 °C. After that, the samples were removed from the distilled water and wiped with tissue paper to remove excess water on the surface of the samples. The final weight (*W*_24*h*_) and thickness (*T*_24*h*_) of the samples were measured immediately. The tests were performed in duplicate. The average values and standard deviations were calculated. The water uptake, and thickness swelling of the biocomposites were calculated based on Formula 1 and 2, respectively,

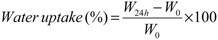
(1)

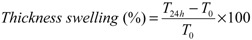
(2)


## 4. Conclusions

In the present work, SHS was successfully employed to modify the surface of OPMF. This treatment increased surface roughness of OPMF due to the removal of impurities and hemicellulose. The tensile test showed that OPMF treated at 220 °C and 60 min produced a biocomposite with optimum tensile properties. It was found that the increment in tensile strength, tensile modulus and elongation at break were 40%, 480% and 26%, respectively. The SEM analysis showed improvement of the interfacial adhesion between SHSOPMF and PBS with formation of a compact solid-like structure. The flexural modulus, flexural strength and impact strength of the biocomposite were enhanced by 45%, 19% and 14%, respectively with the presence of SHSOPMF. Furthermore, the dimensional stability of the SHSOPMF/PBS biocomposite was also improved by 48%. This work demonstrates that SHS offers tremendous potential as a green processing method to be utilized in surface treatment of fiber for biocomposite production.
